# Effectiveness and safety of prophylactic abdominal aortic balloon occlusion for patients with type III caesarean scar pregnancy: a prospective cohort study

**DOI:** 10.1186/s12884-023-06065-8

**Published:** 2023-10-25

**Authors:** Jie Wu, Ruixia Guo, Lixin Li, Danxia Chu, Xinyan Wang

**Affiliations:** Department of Obstetrics and Gynecology, The First Affiliated Hospital of Zhengzhou University, No. 1 East Construction Rd, Zhengzhou, 450052 Henan China

**Keywords:** Caesarean scar pregnancy, Abdominal aortic balloon occlusion, Uterine artery embolization, Effectiveness, Safety

## Abstract

**Background:**

Caesarean scar pregnancy (CSP) is a special type of ectopic pregnancy with a high risk of massive haemorrhage. Few studies have focused on the efficacy of prophylactic abdominal aortic balloon occlusion as a minimally invasive method in caesarean section. This study aimed to evaluate the effectiveness and safety of prophylactic abdominal aortic balloon occlusion for patients with type III CSP.

**Methods:**

This was a prospective cohort study. Patients with type III CSP in the First Affiliated Hospital of Zhengzhou University from January 2020 to June 2022 were enrolled. Eligible patients received prophylactic abdominal aortic balloon occlusion (defined as the AABO group) or uterine artery embolization (defined as the UAE group) before laparoscopic surgery. Clinical outcomes included intraoperative blood loss, body surface radiation dose, hospitalization expenses, and time to serum β-hCG normalization, and safety were also assessed.

**Results:**

A total of 68 patients met the criteria for the study, of whom 34 patients were in the AABO group and 34 patients were in the UAE group. The median intraoperative blood loss in the AABO and UAE groups was 17.5 (interquartile ranges [IQR]: 10, 45) and 10 (IQR: 6.25, 20) mL, respectively (*P* = 0.264). The body surface radiation dose of the AABO group was much lower than that of the UAE group (5.22 ± 0.44 vs. 1441.85 ± 11.59 mGy, *P* < 0.001). The AABO group also had lower hospitalization expenses than the UAE group (2.42 ± 0.51 vs. 3.42 ± 0.85 *10^5 yuan, *P* < 0.001). The average time to serum β-hCG normalization in the AABO group was 28.9 ± 3.21 d, which was similar to that in the UAE group (30.3 ± 3.72 d, *P* = 0.099). In addition, the incidence of adverse events in the AABO group was lower than that in the UAE group (5.9% vs. 58.8%, *P* < 0.001).

**Conclusion:**

Prophylactic AABO was equally as effective as UAE in patients with type III CSP but was safer than UAE during and after the operation.

## Introduction

Caesarean scar pregnancy (CSP) is characterized by the presence of a gestational sac embedded in the scar from a prior caesarean delivery [1]. The prevalence of CSP ranges from approximately 1:2000 to 1:1800 in early pregnancy [2], and CSP accounts for 6.1% of all ectopic pregnancies in women who have had a prior caesarean section [3]. CSP can be divided into three types (type I, type II, and type III) according to the 2016 Chinese Expert Opinion [4]. Type III CSP was defined as gestational sacs that were completely implanted in the myometrium of the uterine scar, protruding towards the bladder or forming an amorphous mass with rich vascularity in the caesarean scar, with a myometrium between the gestational sac and the bladder measuring less than 3 mm in thickness. Type III CSP increases the risks of uterine rupture, massive haemorrhage, hysterectomy, and maternal mortality [5].

Termination of CSP is required. A variety of therapeutic strategies have been described for managing CSP, including drugs, uterine artery embolization (UAE), curettage, and laparoscopic or transvaginal surgery [6]. Haemostasis and fertility preservation are the main objectives of CSP management. According to the Chinese Expert Opinion, patients with type III CSP are prone to massive bleeding or even uterine rupture in early pregnancy, which is extremely dangerous, especially for mass-type CSP [4]. Due to the risk of severe intraoperative blood loss, prophylactic interventional therapy is the most effective measure, followed by sonoscopic surgery [7].

Increasing evidence suggests that UAE can be minimally invasive in CSP in that it can be performed with laparoscopy, hysteroscopy, or local resection [8, 9]. UAE preconditioning can effectively block blood vessels, thus greatly reducing the risk of bleeding and preserving normal reproductive function [10]. However, the incidence of adverse events is reported to be 8.0–51.7% [11], commonly including large-area uterine necrosis, postoperative pain, bladder or rectum necrosis, premature ovarian failure, and postembolization amenorrhea [12]. Abdominal aortic balloon occlusion (AABO), an important method to temporarily and mechanically occlude the blood supply, has been widely used to stop massive bleeding in obstetric surgery [13, 14]. Studies have shown that prophylactic AABO can effectively control intraoperative and postoperative blood loss and reduce the risk of adverse events in patients with placenta previa who are planned for caesarean Sects. [15–17]. However, there is a lack of medical evidence supporting the effectiveness and safety of UAE and AABO for patients with type III CSP. An evidence-based comparison between AABO and UAE may aid proper selection of pretreatment methods for patients with CSP.

This study aimed to evaluate the effectiveness and safety of prophylactic AABO and UAE for patients with type III CSP.

## Methods

### Study design

This was a prospective cohort study. Patients with CSP were enrolled from January 2020 to June 2022 at the First Affiliated Hospital of Zhengzhou University. The inclusion criteria were as follows: (1) aged 18 years or older; (2) 3–16 weeks of gestation; (3) diagnosis and confirmation of type III CSP (described as the presence of a gestational sac completely implanted in the myometrium of the uterine scar and protruding towards the bladder, and the myometrium between the gestational sac and the bladder is ≤ 3 mm in thickness or missing) by both transvaginal colour Doppler sonography (TVCDS) and MRI. The exclusion criteria were as follows: (1) presence of severe vaginal bleeding (emergency); (2) previous history of uterine surgery other than caesarean section; (3) unsuitable conditions making the patient ineligible for hysteroscopic surgery, including bleeding disorder and impaired renal or hepatic function; and (4) refusal to participate in the study. Eligible patients were assigned to the AABO group or the UAE group based on the preconditioning regimen they received.

This study was approved by the institutional review board of the ethics committee of the First Affiliated Hospital of Zhengzhou University (2021-KY-0209-004). Written informed consent forms were obtained from each enrolled patient.

### Procedure

All patients underwent standardized procedures to resolve the caesarean section scar pregnancy. All patients underwent laparoscopic removal of gestational tissues and repair of uterine caesarean scar defects after prophylactic UAE or AABO. Balloon tamponade and UAE were performed by the same radiologist and the same assistants. All operations were performed by the same surgeon and the same surgical assistants and nurses using endoscopic instruments.

**Fig. 1:**
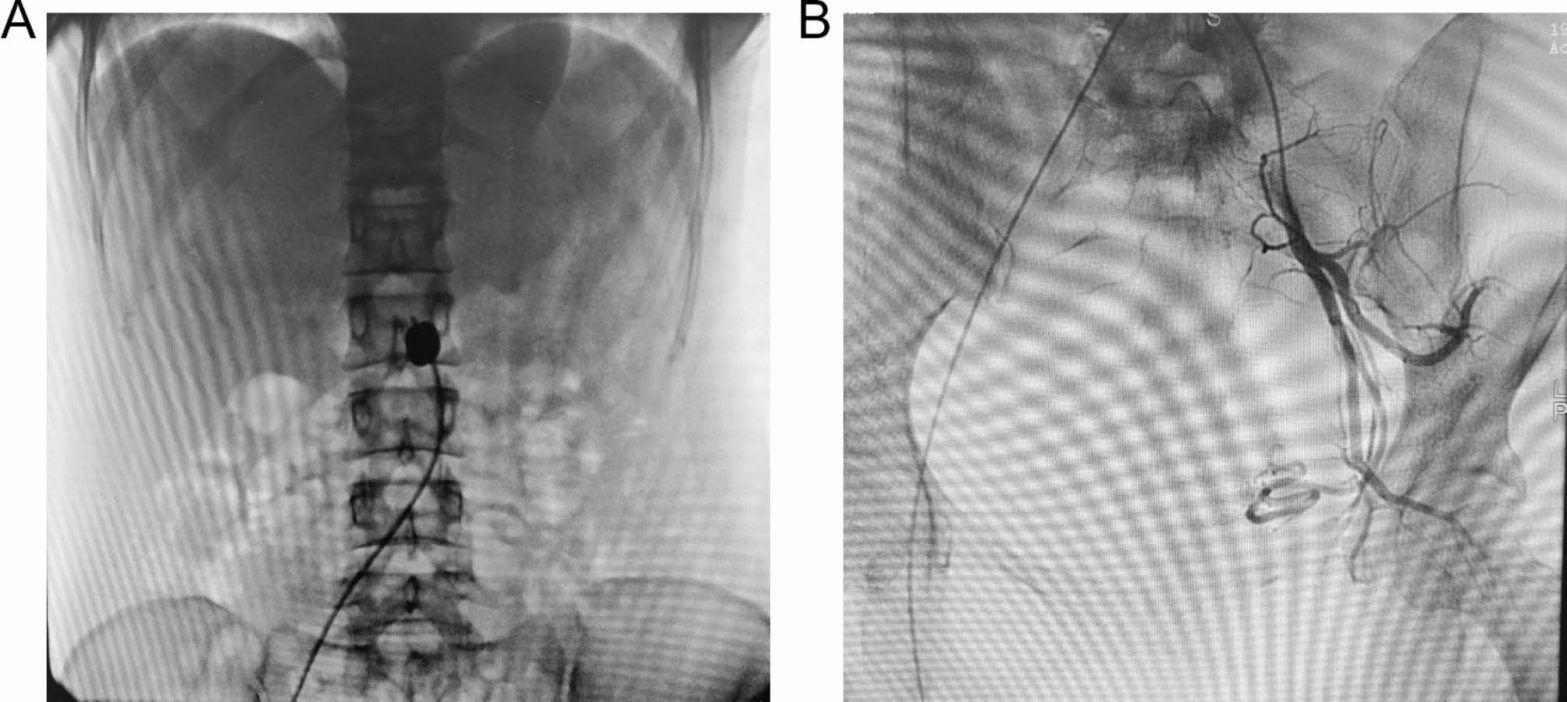
Representative images from the two groups. (**A**) abdominal aortic balloon occusion (OOAB); (**B**) uterine artery embolization (UAE).

Patients with type III CSP in the AABO group were treated with abdominal aortic balloon occlusion prepositioning under digital subtraction angiography (DSA) while undergoing hysteroscopy and negative pressure suction. Under local anaesthesia, femoral artery puncture was performed, an 8 F sheath tube was inserted, and abdominal aortography was performed through the sheath tube. The morphology of the abdominal aorta was clear, and the openings of the double renal arteries and the bifurcation of the common iliac arteries were defined. A 14 mm*10 mm Fogarty balloon catheter was placed into the sheath and inserted into the abdominal aorta below the opening of the renal artery, and the artery sheath and balloon catheter were fixed. The occlusion was observed after localization. The balloon was filled with diluted contrast agent, and abdominal aortic angiography was performed in parallel to show complete occlusion of abdominal aortic blood flow and smooth blood flow into the bilateral renal arteries. The focus was removed under hysteroscopy or laparoscopy immediately after the operation. During the operation, the balloon was filled intermittently to block the abdominal aorta blood flow. The balloon was filled for no more than 30 min at more than 10 min intervals [18]. After the operation, the balloon was withdrawn, the femoral artery puncture site was sutured and pressurized for 12 h, and the lower limb was immobilized for 6 h. Figure 1A shows the AABO.

Patients with type III CSP in the UAE group were treated under DSA. Bilateral internal iliac artery and uterine artery angiography procedures were performed through unilateral femoral artery puncture. Catheters were inserted into the bilateral uterine arteries superselectively, and an appropriate amount of gelatine sponge granules was embolized into the peripheral vessels of the bilateral uterine artery. After embolization, the femoral artery puncture site was sutured and then pressure bandaged for 24 h. Hysteroscopy or laparoscopy combined with debridement was performed 1–3 days after uterine artery embolization. Figure 1B shows the UAE.

### Follow-up

The serum β-human chorionic gonadotropin (β-hCG) level was monitored weekly until it returned to a normal level (defined as a level of less than or equal to 5 mIU/mL). The serum levels of follicle-stimulating hormone (FSH), luteinizing hormone (LH) and oestradiol (E2) were assessed six months after surgery.

### Outcomes

The clinical outcomes included intraoperative blood loss, time under DSA, body surface radiation dose, duration of surgery, postoperative serum β-hCG level, hospitalization expenses, duration of hospital stay, time to serum β-hCG normalization, and safety. The serum levels of FSH, LH, and E2 six months after surgery were also included.

The assessment of adverse events included the occurrence of postoperative postembolization syndrome and decreased menstruation or amenorrhea six months after surgery. Postembolization syndrome was defined by the occurrence of pelvic pain, nausea, vomiting, fever, and perineal swelling [19]. Decreased menstruation is defined as a reduction in the total menstrual volume to less than half of the preoperative volume. Severe adverse events included massive haemorrhage (> 500 mL) and/or hysterectomy.

### Statistical analysis

The data analysis was conducted by IBM SPSS software version 26.0 (IBMCorp., Armonk, N.Y., USA). Continuous variables with normal distribution were reported as the mean ± standard deviation and compared by Student’s t tests between two groups. Continuous variables with nonnormal distributions were expressed as medians (interquartile ranges [IQR]) and compared by the Mann‒Whitney U test. Categorical variables are reported as numbers (%) and were compared by the chi-square test or Fisher’s exact test. A *P* value < 0.05 was considered statistically significant.

## Results

### Patients and baseline characteristics

**Fig. 2:**
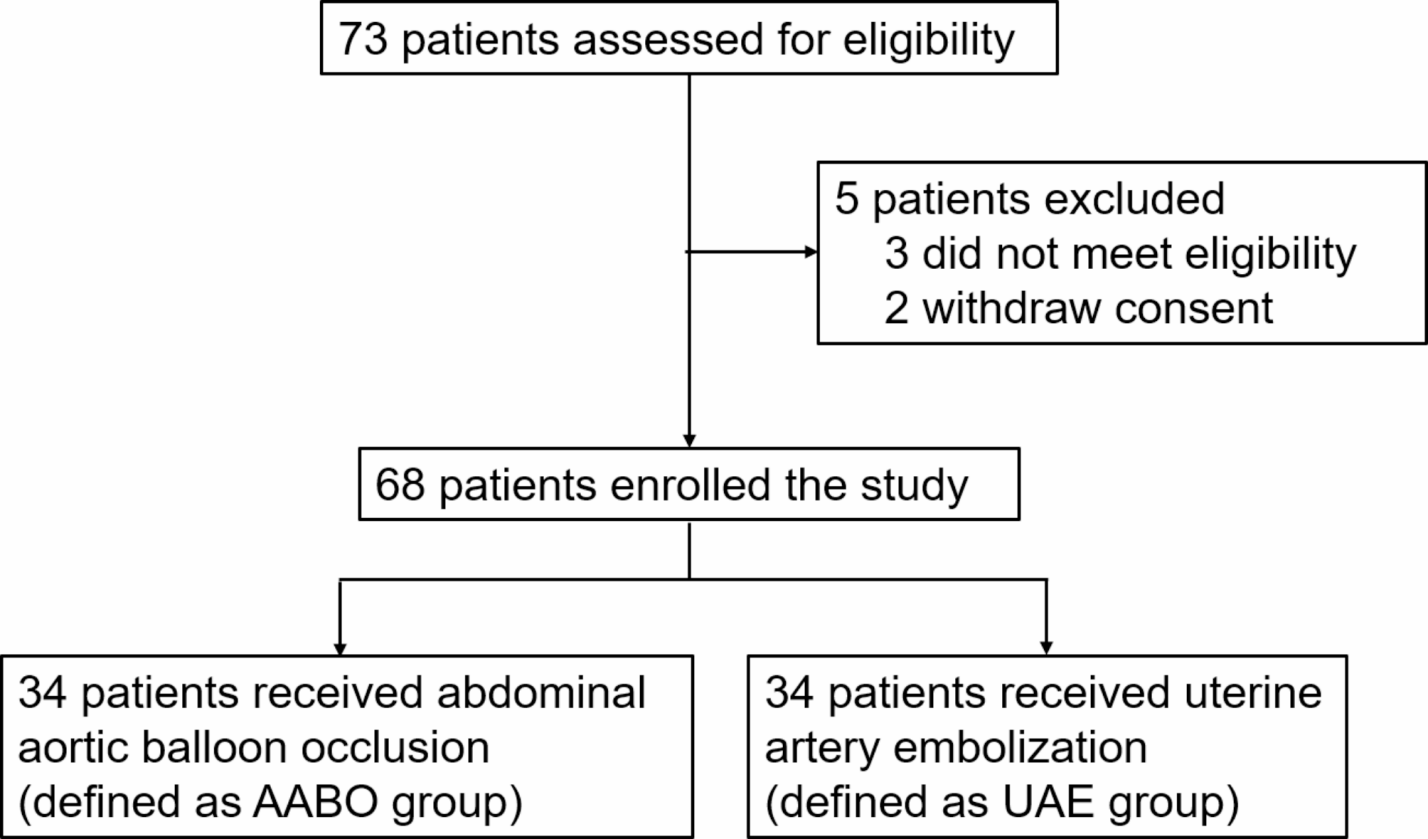
Flowchart of study

A total of 73 patients with a confirmed diagnosis of type III CSP between January 2020 and June 2022 were enrolled, of whom 5 were excluded. Finally, 68 patients were enrolled and randomized in a 1:1 ratio to the AABO group (34 patients) or the UAE group (34 patients) (Fig. 2).

The baseline characteristics of the two groups were well balanced (Table 1). The average age of the patients was 33.79 ± 4.63 years in the AABO group and 32.59 ± 4.32 years the UAE group. Fifteen patients (44.1%) in the AABO group and 11 patients (32.4%) in the UAE group were older than 35 years of age. The median β-hCG levels before treatment were 35,805 (IQR: 19760.5, 69831.5) and 34,175 (IQR: 12,320, 61,221) in the AABO and UAE groups, respectively. There was no difference in the diameter of the gestational sac, length of the embryonic bud, or embryonic heart rate (Table 1).

**Table 1:** Baseline characteristics

	AABO group (n = 34)	UAE group (n = 34)	*P*
Age, n (%)			
< 35 years	19 (55.9)	23 (67.6)	0.318
≥ 35 years	15 (44.1)	11 (32.4)	
Gestational age (weeks)	7.3 (6.5, 9.4)	7.3 (6.3, 8.0)	0.332
Body mass index (kg/m^2^)	24.0 (22.1, 26.8)	24.1 (22.2, 25.5)	0.695
Gravidity*, n (%)			0.808
≤ 4	19 (55.9)	18 (52.9)	
> 4	15 (44.1)	16 (47.1)	
Parity, n (%)			0.595
1	11 (32.4)	9 (26.5)	
> 1	23 (67.6)	25 (73.5)	
Prior cesarean section, n (%)			1.000
1	14 (41.2)	14 (41.2)	
> 1	20 (58.8)	20 (58.8)	
Time interval from previous cesarean section (m)	36.6 (30, 52.2)	38.4 (27.6, 55.2)	0.749
β-HCG before treatment (mIU/mL)	35,805 (19760.5, 69831.5)	34,175 (12,320, 61,221)	0.806
Gestational sac diameter (mm)	24.5 (20.0, 26.0)	24.0 (20.0, 25.0)	0.492
Embryonic bud length (mm)	10.0 (9.0, 12.0)	10.0 (9.0, 12.0)	0.533
Embryonic heartbeat, n (%)			
Yes	34 (100)	34 (100)	/

* Includes the current caesarean section pregnancy

### Intraoperative conditions

The operation was successful in both groups. The median intraoperative blood loss volume in the AABO and UAE groups was 17.5 (IQR: 10, 45) and 10 (IQR: 6.25, 20) mL, respectively (*P* = 0.264). The duration of surgery in the AABO group and UAE group was 35.5 (IQR: 23.25, 50.75) min and 31.5 (IQR: 20, 54.25) min, respectively (*P* = 0.300). The time under DSA in the AABO group was significantly lower than that in the UAE group (7 [IQR: 6, 7.75] vs. 1140 [IQR: 1097.75, 1160)] s, *P* < 0.001). The body surface radiation dose in the AABO group was much lower than that in the UAE group (5.22 ± 0.44 vs. 1441.85 ± 11.59 mGy, *P* < 0.001). (Table 2).

**Table 2:** Intraoperative and postoperative conditions of AABO and UAE

	AABO group (n = 34)	UAE group (n = 34)	*P*
**Intraoperative**			
Intraoperative blood loss (mL)	17.5 (10, 45)	10 (6.25, 20)	0.264
Time under DSA (s)	7 (6, 7.75)	1140 (1097.75, 1160)	< 0.001
Body surface radiation dose (mGy)	5.22 ± 0.44	1441.85 ± 11.59	< 0.001
Durations of surgery (min)	35.5 (23.25, 50.75)	31.5 (20, 54.25)	0.300
**Postoperative**			
Postoperative serum β-hCG (mIU/mL)	6612.5 (3103.75, 9996.25)	4615 (954.175, 12,897)	0.581
Length of hospital stay (d)	2.5 (2, 3)	4 (3, 5)	< 0.001
Time for serum β-hCG normalization (d)	28.9 ± 3.21	30.3 ± 3.72	0.099

### Postoperative conditions

There was no significant difference in the β-hCG levels between the AABO group and the UAE group on Day 2 after surgery (7759.65 ± 6730.79 vs. 6996.20 ± 6744.51 mIU/mL, *P* = 0.581). The duration of hospital stay was 2.5 (IQR: 2, 3) and 4 (IQR: 3, 5) days, respectively (*P* < 0.001). The hospitalization expenses were also significantly lower in the AABO group than in the UAE group (2.42 ± 0.51 vs. 3.42 ± 0.85 *10^5 yuan, *P* < 0.001). The time to serum β-hCG normalization in the AABO group was 28.9 ± 3.21 d, which was similar to that in the UAE group (30.3 ± 3.72 d, *P* = 0.099) (Table 2).

### Ovarian function

The serum FSH, LH, and E2 levels were measured at six months after surgery in both the AABO and UAE groups. In the AABO group, the FSH, LH and E2 levels were 78.84 (IQR: 38.38, 117.58) U/L, 7.96 (IQR: 4.0, 12.76) U/L and 5.63 (IQR: 4.37, 8.38) pmol/L, respectively. In the UAE groups, the FSH, LH and E2 levels were 66.1 (IQR: 28.46, 113.63) U/L, 8.07 (IQR: 6.67, 12.21) U/L and 6.14 (IQR: 4.09, 7.80) pmol/L, respectively. There were no statistically significant differences between the two groups.

### Safety

The incidence of adverse events was 5.9% in the AABO group, which was less than that in the UAE group (58.8%, *P* < 0.001). No serious adverse effects occurred during treatment or follow-ups. None of the patients showed any sign of massive vaginal bleeding during or after surgery, and no hysterectomy was performed. In the AABO group, one patient experienced postembolization syndrome. In the UAE group, 16 patients had postembolization syndrome, including two patients with lower abdominal pain, 10 patients with hip pain, two patients with fever, and two patients with nausea and vomiting (Table 3). Decreased menstruation was observed in one patient (2.9%) in the AABO group and in four patients (11.8%) in the UAE group. None of the patients developed amenorrhea after surgery.

**Table 3:** Safety of AABO and UAE

	AABO group (n = 34)	UAE group (n = 34)	*P*
Any adverse events, n (%)	2 (5.9)	20 (58.8)	< 0.001
Serious adverse events, n (%)	0	0	
Post-embolization syndrome, n (%)	1 (2.9)	16 (47.1)	
Abdominal pain	0	2 (5.9)	
Hip pain	0	10 (29.4)	
Fever	1 (2.9)	2 (5.9)	
Nausea and vomiting	0	2 (5.9)	
Decreased menstruation, n (%)	1 (2.9)	4 (11.8)	
Amenorrhea, n (%)	0	0	

## Discussion

CSP is considered a special type of ectopic pregnancy that must be terminated once diagnosed due to the increased risk of fatal bleeding. Studies have shown that prophylactic treatment can significantly reduce the risk of intraoperative haemorrhage in patients with type III CSP. However, there is no standardized treatment for type III CSP. In this study, 68 patients with type III CSP were treated with AABO or UAE as a prophylactic treatment. The results showed that the prophylactic AABO and UAE both are effective methods for patients with type III CSP, but AABO is safer and has fewer complications than UAE.

Because the abnormally attached placenta has an extremely rich supply of blood vessels, bleeding during the operation is potentially fatal. In addition, due to difficult exposure of the operating field and the risk of massive bleeding, emergency haemostasis is difficult intraoperatively. Reducing the incidence of intraoperative bleeding and ensuring rapid haemostasis are essential to the successful rescue of placental attachment. Many studies have shown that patients with severe CSP III should undergo UAE to significantly decrease the risk of intraoperative haemorrhage [20, 21]. In our study, the median intraoperative blood loss volume was 17.5 mL and 10 mL for AABO and UAE, respectively, thereby strongly indicating that both methods can reduce intraoperative bleeding and preserve the uterus. The operation time of the balloon group was slightly longer than that of the embolization group due to the repeated release of the balloon, observation of bleeding points, and haemostasis during the operation. However, there was no statistical significance between the two groups. In the AABO group, the balloon was released several times during the operation to show the bleeding site. The main uterine artery and each branch maintained unobstructed blood flow, and the uterine body blood supply was unaffected. Balloon occlusion is a temporary means of mechanical occlusion of the blood supply, as the balloon is intravascularly precisely transported under DSA guidance. This method helps to reduce intraoperative bleeding [22]. At present, abdominal aortic balloon occlusion in obstetric and gynaecologic surgeries blocks most of the pelvic blood supply, thereby effectively controlling bleeding in the abdominal aortic plane [17]. In addition, previous studies have shown that balloon dilation time should not be prolonged so as to avoid thrombotic complications [23]. Otherwise, ischaemia‒reperfusion injury may affect the pelvic organs, lower limbs, kidneys, and spinal cord [24]. If prolonged or continuous arterial occlusion is required during the operation, balloon occlusion should be stopped intermittently to restore the blood supply. In addition, arterial thrombosis is also one of the most important complications, with an incidence rate of 5% [25].

There was a previous study raising concerns for potential radiation injury in both the patient and foetus during AABO [26]. However, in this study, the intraoperative time of AABO under DSA was only 3–8 s, which was much faster than the operation time of UAE. It is generally believed that radiation has adverse effects on female reproduction, leading to miscarriage and congenital abnormalities [27]. AABO does not require prolonged exposure or occupational exposure to ionizing radiation in medical care. The average length of hospital stay was shorter and the total hospitalization expenses were lower in the AABO group, which may be related to the lower incidence of postoperative complications and the lower cost of abdominal aortic balloon supplies than in the UAE group. Similarly, Guo et al. found that the UAE group had a significantly longer hospital stay and a higher total cost of hospitalization [8]. This is consistent with our findings. The time to β-hCG normalization in the AABO group was slightly slower than that in the UAE group, but the difference was not significant. A meta-analysis showed that the time to serum β-hCG normalization was approximately 30.3 days in the UAE group [28], which was similar to our results.

In this study, ovarian function was mainly assessed through sex hormone detection. Ovarian function in the patients in the two interventional treatment groups was comparable, and AABO presented a reduced risk of amenorrhea and infertility postoperatively. UAE is believed to cause ovarian ischaemia and decreased ovarian function. The cause for this may be uterine artery ovarian branch blockage or diffusion of embolization material into the ovarian vessels through the uterine artery, followed by partial vessel plugging [29]. In addition, randomized trials have suggested that ovarian function may decrease following UAE in patients over the age of 45 years old [30].

In our study, of the patients who underwent UAE, 10 complained of hip pain, and four suffered from decreased menstruation, with a total complication rate of 58.8%. Adverse events associated with UAE have been reported extensively. The overall incidence of postoperative complications ranged from 17.6 to 23% in the UAE pretreatment group [8, 31]. A recent review by Timor-Tritsch [32] reported that UAE treatment results in increased rates of fever, as well as mild pain in the abdomen or the pelvic region, with an overall complication rate of 46.9%. Cao et al. [12] followed 119 patients with CSP who underwent UAE to assess their menstrual recovery and their rate of uterine curettage. Among these patients, 58 (57.4%) had hypomenorrhea, and 2 (1.7%) had amenorrhea. Tang et al. [33] reported that fifty-five patients (1.2%) managed by UAE had severe complications. Our results suggested that patients who underwent AABO had significantly fewer complications than those who underwent UAE. Here, the data indicated that patients who underwent prophylactic AABO had significantly fewer complications than those who underwent UAE, suggesting that AABO may be suited to be used as a pretreatment for type III CSP.

Despite some significant findings that AABO is a reliable treatment method for type III CSP, especially when compared with UAE, there are still some limitations in this study. First, the small sample size is a major limitation. Large-scale clinical studies are needed to support our conclusions. Second, the sudden changes in total peripheral resistance caused by balloon occlusion and opening during abdominal aorta occlusion and reperfusion injury are likely to cause haemodynamic disorders, and markers of blood reperfusion injury should be evaluated after surgery. However, no further studies have been conducted on the haemodynamic changes of the two interventional methods due to lack of the patient acceptance.

## Conclusions

Both AABO and UAE are effective prophylactic methods for patients with type III CSP. In comparison to UAE, AABO is safe and has few complications. The effectiveness and safety of AABO should be confirmed in larger scale studies in the future.

## Data Availability

The datasets used for analysis during the current study are available from the corresponding author on request.

## Abbreviations

CSP: cesarean scar pregnancy
UAE: uterine artery embolization
AABO: abdominal aortic balloon occlusion
TVCDS: trans-vaginal color doppler sonography
DSA: digital subtraction angiography
β-hCG: β-human chorionic gonadotropin
FSH: follicle-stimulating hormone
LH: luteinizing hormone
E2: estradiol
IQR: interquartile ranges

## References

[1] Miller R, Gyamfi-Bannerman C (2022). Society for maternal-fetal Medicine Consult Series #63: cesarean scar ectopic pregnancy. Am J Obstet Gynecol.

[2] Rotas MA, Haberman S, Levgur M (2006). Cesarean scar ectopic pregnancies: etiology, diagnosis, and management. Obstet Gynecol.

[3] Seow KM, Huang LW, Lin YH (2004). Cesarean scar pregnancy: issues in management. Ultrasound Obstet Gynecol.

[4] [Expert opinion of diagnosis and treatment of cesarean scar pregnancy. (2016)]. *Zhonghua Fu Chan Ke Za Zhi*. 2016;51:568–572. 10.3760/cma.j.issn.0529-567X.2016.08.003. 10.3760/cma.j.issn.0529-567X.2016.08.003 27561933

[5] Glenn TL, Bembry J, Findley AD (2018). Cesarean scar ectopic pregnancy: current management strategies. Obstet Gynecol Surv.

[6] Birch Petersen K, Hoffmann E, Rifbjerg Larsen C (2016). Cesarean scar pregnancy: a systematic review of treatment studies. Fertil Steril.

[7] Polat I, Ekiz A, Acar DK (2016). Suction curettage as first line treatment in cases with cesarean scar pregnancy: feasibility and effectiveness in early pregnancy. J Matern Fetal Neonatal Med.

[8] Guo J, Yu J, Zhang Q (2018). Clinical efficacy and safety of uterine artery embolization (UAE) versus laparoscopic cesarean scar pregnancy debridement Surgery (LCSPDS) in treatment of cesarean scar pregnancy. Med Sci Monit.

[9] Wang J, Zhao R, Qian H (2021). Pituitrin local injection versus uterine artery embolization in the management of cesarean scar pregnancy: a retrospective cohort study. J Obstet Gynaecol Res.

[10] Gu Z, Jia P, Gao Z (2022). Uterine artery embolization combined with ultrasound-guided dilation and curettage for the treatment of cesarean scar pregnancy: efficacy and 5-8-year follow-up study. J Interv Med.

[11] Liang F, He J (2010). Methotrexate-based bilateral uterine arterial chemoembolization for treatment of cesarean scar pregnancy. Acta Obstet Gynecol Scand.

[12] Cao GS, Liu RQ, Liu YY (2018). Menstruation recovery in scar pregnancy patients undergoing UAE and curettage and its influencing factors. Med (Baltim).

[13] Zheng W, Dou R, Yan J (2022). Intra-abdominal aortic balloon occlusion in the management of placenta percreta. Chin Med J (Engl).

[14] Yin H, Hu R (2022). Outcomes of prophylactic abdominal aortic balloon occlusion in patients with placenta previa accreta: a propensity score matching analysis. BMC Pregnancy Childbirth.

[15] Huo F, Liang H, Feng Y (2021). Prophylactic temporary abdominal aortic balloon occlusion for patients with pernicious placenta previa: a retrospective study. BMC Anesthesiol.

[16] Liu C, Yang DD, Qu HB (2021). Efficacy and safety of prophylactic abdominal aortic balloon occlusion versus internal iliac arterial balloon occlusion for placenta accreta spectrum disorder: a systematic review and meta-analysis. Clin Imaging.

[17] Zhang JH, Duan HJ, Zhao YP (2020). Preliminary study on the application of abdominal aortic balloon occlusion in the treatment of cesarean scar pregnancy. Zhonghua Fu Chan Ke Za Zhi.

[18] Bodner LJ, Nosher JL, Gribbin C (2006). Balloon-assisted occlusion of the internal iliac arteries in patients with placenta accreta/percreta. Cardiovasc Intervent Radiol.

[19] Rajan DK, Beecroft JR, Clark TW (2004). Risk of intrauterine infectious Complications after uterine artery embolization. J Vasc Interv Radiol.

[20] Hong Y, Guo Q, Pu Y (2017). Outcome of high-intensity focused ultrasound and uterine artery embolization in the treatment and management of cesarean scar pregnancy: a retrospective study. Med (Baltim).

[21] Ma Y, Yang C, Shao X (2017). Efficacy comparison of transcatheter arterial embolization with gelatin sponge and polyvinyl alcohol particles for the management of cesarean scar pregnancy and follow-up study. J Obstet Gynaecol Res.

[22] Lu YM, Guo YR, Zhou MY (2020). Indwelling Intrauterine Foley Balloon Catheter for Intraoperative and postoperative bleeding in cesarean scar pregnancy. J Minim Invasive Gynecol.

[23] Luo Y, Duan H, Liu WL (2013). Clinical evaluation for lower abdominal aorta balloon occluding in the pelvic and sacral Tumor resection. J Surg Oncol.

[24] Saito N, Matsumoto H, Yagi T (2015). Evaluation of the safety and feasibility of resuscitative endovascular balloon occlusion of the aorta. J Trauma Acute Care Surg.

[25] Teare J, Evans E, Belli A (2014). Sciatic nerve ischaemia after iliac artery occlusion balloon catheter placement for placenta percreta. Int J Obstet Anesth.

[26] Wei LC, Gong GY, Chen JH (2018). Application of lower abdominal aorta balloon occlusion technique by ultrasound guiding during caesarean section in patients with pernicious placenta previa. Zhong Hua Yi Xue Za Zhi.

[27] Kumar S, Sharma A, Kshetrimayum C (2019). Environmental & occupational exposure & female reproductive dysfunction. Indian J Med Res.

[28] Marchand GJ, Masoud AT, Coriell C, et al. Treatment of cesarean scar ectopic pregnancy in China with Uterine Artery Embolization-A Systematic Review and Meta-analysis. J Clin Med. 2022;11. 10.3390/jcm11247393. 10.3390/jcm11247393 PMC9783593 36556010

[29] Czuczwar P, Stepniak A, Milart P (2018). Comparison of the influence of three fibroid treatment options: supracervical hysterectomy, ulipristal acetate and uterine artery embolization on ovarian reserve - an observational study. J Ovarian Res.

[30] Kaump GR, Spies JB (2013). The impact of uterine artery embolization on ovarian function. J Vasc Interv Radiol.

[31] Zhao Q, Sun XY, Ma SQ (2022). Temporary Internal Iliac artery blockage versus uterine artery embolization in patients after laparoscopic pregnancy tissue removal due to cesarean scar pregnancy. Int J Gen Med.

[32] Timor-Tritsch IE, Monteagudo A (2012). Unforeseen consequences of the increasing rate of cesarean deliveries: early placenta accreta and cesarean scar pregnancy. A review. Am J Obstet Gynecol.

[33] Tang F, Du S, Zhao Y (2019). Clinical analysis of uterine artery embolization combined with double balloon catheter plus curettage for patients with placenta previa who underwent pregnancy termination and suffered antenatal massive Hemorrhage in the 2nd trimester: three case reports. Med (Baltim).

